# Age and the risk of All-Terrain Vehicle-related injuries in children and adolescents: a cross sectional study

**DOI:** 10.1186/s12887-017-0807-y

**Published:** 2017-03-17

**Authors:** Lianne McLean, Kelly Russell, Steven McFaull, Lynne Warda, Milton Tenenbein, Jonathan McGavock

**Affiliations:** 1 Children’s Hospital of Winnipeg, The Department of Paediatrics and Child Health, Faculty of Medicine, The University of Manitoba, Winnipeg, MB Canada; 2 The Children’s Hospital Research Institute of Manitoba, Winnipeg, MB Canada; 3 The Public Health Agency of Canada, Ottawa, Ontario Canada; 4 Department of Pediatrics and Child Health, Faculty of Medicine, University of Manitoba, 511 JBRC. Children’s Hospital Research Institute of Manitoba, 715 McDermot Ave., Winnipeg, MB R3E 3P4 Canada

**Keywords:** All-terrain vehicles, Serious injuries, Head injuries, Policy, Children

## Abstract

**Background:**

The study was designed to determine if youth <16 years are at a greater risk of serious injuries related to all-terrain vehicle (ATV) use compared to older adolescents and adults.

**Methods:**

We performed cross sectional study of children and adults presenting to pediatric and adult emergency departments between 1990 and 2009 in Canada. The primary exposure variable was age <16 years and the primary outcome measure was moderate to serious injury determined from physician report of type and severity of injury.

**Results:**

Among 5005 individuals with complete data, 58% were <16 years and 35% were admitted to hospital. The odds of a moderate to serious injury versus minor injury among ATV users <16 years of age was not different compared with those ≥16 years of age (OR: 0.94; 95% CI: 0.84, 1.06). After adjusting for era, helmet use, sex and driver status, youth <16 years were more likely to present with a head injury (aOR: 1.45; 95% CI: 1.19–1.77) or fractures (aOR: 1.60; 95% CI: 1.43–1.81), compared with those ≥16 years. Male participants (aOR: 1.21; 95% CI: 1.06–1.38) and drivers (aOR: 1.30, 95% CI: 1.12–1.51) were more likely to experience moderate or serious injuries than females and passengers. Helmet use was associated with significant protection from head injuries (aOR: 0.59; 95% CI: 0.44–0.78).

**Conclusions:**

Youth under 16 years are at an increased risk of head injuries and fractures. For youth and adults presenting to emergency departments with an ATV-related injury, moderate to serious injuries associated with ATV use are more common among drivers and males. Helmet use protected against head injuries, suggesting minimum age limits for ATV use and helmet use are warranted.

## Background

All-terrain vehicle (ATV) related injuries increased significantly during the past two decades in North America [1–3]. In the United States, youth under 16 years of age represented 23% of all ATV-related deaths between 2000 and 2007 [4] and ~50,000 youth present to emergency departments with an ATV-related injury annually [5, 6]. Annual rates of ATV injury among youth under 15 years of age in the United States over the past decade are between 42-67/100,000 [7]. The 10-year health care cost associated with ATV-related injuries is $1.1 billion USD [8]. With the increasing incidence of ATV-related injuries and the associated treatment costs, public health strategies are needed to reduce the burden of the ATV-related injuries among youth.

In response to an increase in ATV-related injuries and deaths in youth, the Canadian Paediatric Society [1] and the American Academy of Pediatrics [9] published position statements encouraging legislation for a minimum age for ATV use. Factors cited to support their position include increasing rates of ATV-related injuries among youth under 16 years of age and the assumption that younger children are at a greater risk for more severe injuries due to the mismatch in size of the child and weight of the vehicles [1]. Studies in the US suggest that children 12–15 years are more likely to die [10] or be hospitalized [7] from an ATV-related injury compared with those less than 16 years of age. Despite a growing body of literature related to ATV-related injuries in youth [1, 7, 10–15], to the best of our knowledge, none have been designed specifically to determine if the risk of moderate to serious injuries is higher in younger age groups, compared to adults or older adolescents [7, 11]. Using cross sectional data collected between 1990 and 2009 in Canadian emergency departments, we tested the hypothesis that the odds of moderate to serious ATV-related injuries will be greater among youth under 16 years of age compared with those 16 years of age and older. A secondary aim was to describe the primary determinants of several types of ATV-related injuries (moderate to serious, head injuries and fractures).

## Methods

We conducted a cross sectional study of moderate to serious injuries between youth and older adolescents/adults embedded within the Canadian Hospitals Injury Reporting and Prevention Program (CHIRPP) survey. The surveillance system captures data on all injury related Emergency Department visits at 17 participating centres across most Canadian provinces and one territory [16]. Eligible participants include those who agree to provide information related to the injury and who present to a health care professional that completes a form detailing elements of the injury. For the purposes of this study, surveys were collected from caregiver of a child and the attending physician during a visit to one of 10 pediatric and 4 general emergency departments participating in CHIRPP. We restricted analyses to those injured while riding or driving an ATV between March 5 1990 and December 29 2009 [11, 16] and those who were struck by an ATV were excluded (e.g., walking along a road and hit by an ATV). The patient-completed portion of the survey includes information concerning the mechanism of injury, circumstances surrounding the injury, safety equipment, vehicular involvement, and demographic characteristics. Medical or trained research staff enter codes for up to three injured body parts and three types of injuries. Disposition information regarding admission, discharge, and treatment is also entered by medical or research staff. Forms are collected by the Public Health Agency of Canada for coded entry into a national database. CHIRPP data are publically available (http://www.phac-aspc.gc.ca/injury-bles/chirpp/index-eng.php), and the injury data have been validated and are considered reliable [17]. To test the primary hypothesis, we performed a cross sectional comparison of patients admitted to a CHIRPP Hospital for an ATV injury stratified at 16 years of age to determine if the odds of moderate to serious injuries versus mild injuries were different between the groups. Participants provided verbal consent to participation at the time of the visit to the emergency department. For children under 16 years of age, parents/guardians provided verbal consent and reported on their behalf. The study was approved by the Health Research Ethics Board at the University of Manitoba (HS15719-H2012:311).

### Exposure of interest

The primary exposure of interest was age less than 16 years (herein referred to as youth), with reference group consisting of individuals 16 years and older (herein referred to as adults). with the maximum age surveyed being 93 years old. Because the majority of Canadian provinces have released statements or guidelines prohibiting ATV use by youth less than 16 years of age [1] and 16 is the minimum drivers age in most Canadian provinces, age was stratified at less than 16 years.

### Outcome measures

The primary outcome measure was a composite measure of moderate to serious injuries classified according to the CHIRPP Injury Severity Proxy Scale. Except for intracranial, dental, multiple injuries of more than one nature and fatalities (which are scaled directly from the nature of injury), all injuries are stratified into their final categories (minor, moderate, serious, fatal or unclassifiable) by disposition and, in the case of skull and facial fractures, associated closed head injuries. Mild injuries are those where the patient received treatment in the emergency department, but was not admitted to hospital and, for skull/facial fractures, suffered no associated closed head injuries. Moderate or serious injuries are those where the patient was admitted to hospital or experienced traumatic amputations (i.e. those involving the fingers or toes regardless of disposition), or, in the case of skull/facial fractures, had an associated closed head injury. For youth with multiple injuries, the most serious injury was used to classify injury severity. The injury section at the Public Health Agency of Canada developed the injury severity proxy scale for specific use with CHIRPP.

### Potential confounders

The following potential confounders are hypothesized determinants of ATV injury severity and were extracted from the CHIRPP dataset: helmet use, driver status, sex, region of country where the patient was seen, and the era (stratified in 5 year intervals). A previous study found that boys were more likely to experience ATV-related injuries [5]. Helmet use was included as a co-variate for head injuries, as previous studies [13, 18, 19] have demonstrated the protective effects of helmets when using an ATV. Era was included as rates of ATV-related injuries have varied by era over the past three decades [3]. Because provincial legislation [1] and ATV-related injury rates vary across regions in Canada [20], the role of region (east, west, central) was included in the analyses.

### Statistical analyses

Demographic and injury characteristics were described as proportions and tested for statistical significance using the *χ* ^2^ test. Odds ratios and 95% confidence intervals (CI) were calculated. To test the study hypothesis, we performed unadjusted logistic regression analyses to determine if the odds of a moderate to serious injury were higher among youth than adults. To address the risk of confounding, a logistic regression model was developed using backward elimination techniques that contained all potential confounders (sex, driver status, era, and region) and included interaction terms to explore effect modification by sex and driver versus passenger status. Driver was defined as a self-reported operator of the ATV during the time of the accident. If the omnibus test was not significant (*p* > 0.05), the interaction terms were removed from the model as there was no evidence of effect modification. Co-variates that changed the injury severity estimate by 15% or more were retained in the final model, as they were considered to confound the relationship between injury severity and age. This analysis was repeated when the outcome was severity of head injury and helmet use was included as a potential confounder. Logistic regression was used to determine risk factors for common isolated moderate to serious injuries and these models contained all co-variates. An alpha < 0.05 was considered statistically significant for groupwise comparisons of outcome variables. In order to ensure that the risk factor predicted a particular injury and not a combination of injuries, only those with one isolated injury were included. The final sample size of 5002, was the largest sample available for participants with compete data, that were injured while riding an ATV and information for key confounding were available. As less than 5% of data were missing, we did not impute missing data and no sensitivity analyses were performed. All data were analyzed using Statistical Package for the Social Sciences (SPSS) version 19.0 (Chicago, ILL) and STATA version 12 (College Station, TX).

## Results

### Participant characteristics and types of injuries

**Fig. 1:**
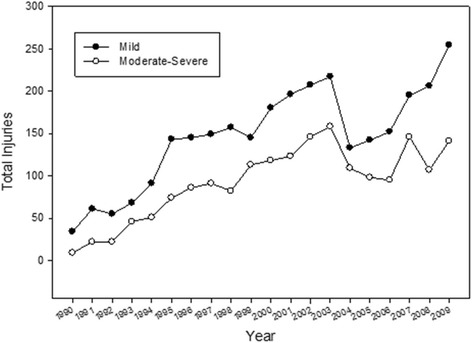
Annual number of ATV-related injuries in the CHIRPP database between 1990-2009

Between 1990 and 2009, 5002 youth and adults presented to an emergency department within CHIRPP network with an injury that occurred while riding an ATV. Youth and adults reporting to emergency departments within the CHIRPP network increased over the 20-year surveillance period (Fig. 1). Of these, 2883 (58%) were youth (<16 years old), 2119 (42%) were adults (≥16 years old), and age was not reported in three instances. Participant characteristics are provided in Table 1.

**Table 1:** Demographic characteristics of 5002 younger and older injured ATV users

Variable	Adults (≥16 yrs)	Youth (< 16 yrs)	p
	*n* = 2119 (42.4%)	*n* = 2883 (57.6%)	
Gender			
Male (%)	1676 (79.1)	2006 (69.6)	< 0.01
Driver Status			
Driver (%)	1426 (67.3)	1224 (42.5)	< 0.001
Helmet Use			
Yes (%)	799 (37.7)	1288 (44.7)	< 0.001
Era of Injury			
1990–1995 (%)	233 (11.0)	460 (16.0)	< 0.001
1996–2000 (%)	510 (24.1)	762 (26.4)	
2001–2005 (%)	661 (31.2)	881 (30.6)	
2006–2010 (%)	715 (33.7)	780 (27.1)	
Region			
Eastern^a^ (%)	669 (31.6)	1110 (38.5)	< 0.001
Central^b^ (%)	1256 (59.3)	1376 (47.7)	< 0.001
Western^c^ (%)	194 (9.2)	397 (13.8)	< 0.001
Admission Status			
Admitted (%)	728 (34.4)	1015 (35.2)	NS
Injury Severity			
Mild (%)	1257 (59.3)	1752 (59.8)	NS
Moderate (%)	430 (20.3)	681 (23.6)	NS
Serious (%)	364 (17.2)	343 (11.9)	< 0.001
Fatal (%)	2 (0.1)	6 (0.2)	

^a^Eastern Region = Centres within provinces of Nova Scotia, Newfoundland and Quebec; ^b^Central Region = Centres within provinces of Manitoba and Ontario and ^c^Western Region = Centres within Saskatchewan, Alberta, British Columbia and the Northwest Territories

*NS* not significantly different from adults

Youth were less likely to be drivers (53 vs 67%, *p* < 0.001) and were more likely to wear helmets (48 vs 38%, *p* < 0.001). Youth constituted a smaller proportion of admissions over the 20-year follow-up period and adults were seen more often in pediatric emergency departments in the central region of Canada, compared with other regions (Table 1). When stratified by injury severity, 60% of injuries were mild, 22% were moderate, 14% were serious, 0.16% were fatal, and 4% were unclassified.

Fractures (39%), superficial wounds (18%), and open wounds (11%) were the three most common injuries among youth (Table 2). The odds of a fracture versus any other type of injury were significantly higher among youth compared with adults (OR: 1.33; 95% CI: 1.20, 1.47). Open wound (OR: 1.30; 95% CI: 1.10, 1.53), head injuries (OR: 1.33; 95% CI: 1.11, 1.59), and crush injuries (OR: 2.13; 95% CI: 1.10, 4.11) were also significantly more common among youth (Table 2). While injuries resulting in amputations were rare, they were more common among youth (*n* = 10) than adults (*n* = 3); however this difference was not statistically significant.

**Table 2:** Description of the 7018 injuries sustained by 5002 ATV users

*Type of Injury*	Total	Adults (≥ 16 yrs)	Youth (< 16 yrs)	Odds Ratio (95% CI)
		*n* ^a^ = 2954 (42.1%)	*n* ^a^ = 4064 (57.9%)	
Fracture	2526 (36.0)	952 (32.2)	1576 (38.7)	**1.33 (1.20, 1.47)**
Superficial	1331 (19.0)	596 (20.2)	735 (18.1)	**0.87 (0.77, 0.99)**
Open wound	699 (10.0)	255 (8.6)	444 (10.9)	**1.30 (1.10, 1.53)**
Soft tissue	642 (9.1)	346 (11.7)	296 (7.3)	**0.59 (0.50, 0.70)**
Brain injury	555 (7.9)	199 (6.7)	356 (8.8)	**1.33 (1.11, 1.59)**
Dislocation/sprain	451 (6.4)	227 (7.7)	224 (5.5)	**0.70 (0.58, 0.85)**
Internal organs	183 (2.6)	79 (2.7)	104 (2.6)	0.96 (0.71, 1.29)
Muscle/tendon	86 (1.2)	48 (1.6)	38 (0.9)	**0.57 (0.37, 0.88)**
Crushing	47 (0.7)	12 (0.4)	35 (0.9)	**2.13 (1.10, 4.11)**
Amputation	13 (0.2)	3 (0.1)	10 (0.3)	2.43 (0.67, 8.82)
Other	260 (3.7)	108 (3.7)	152 (3.7)	1.02 (0.80, 1.32)
Multiple injuries	216 (3.1)	125 (4.2)	91 (2.2)	**0.52(0.39, 0.68)**

Significantly increased or reduced odds are bolded

^a^N refers to number of injuries; Reference group = Adults

### Determinants of injury severity

Characteristics of patients with moderate to serious and mild injuries are provided in Table 3. Compared with mild injuries, patients with moderate to serious injuries were significantly more likely to be male (OR: 1.21; 95% CI: 1.06, 1.38) or driving the ATV at the time of the injury (OR: 1.30; 95% CI: 1.12, 1.51). Moderate to serious injuries were 1.2–1.5 fold more common between 1996 and 2005 compared with other eras and more likely to occur in the Eastern region of Canada. There was no significant difference in the odds of a moderate to serious injury versus minor injury among youth ATV users compared with adult ATV users (crude OR: 0.94; 95% CI: 0.84, 1.06). There was no appreciable difference in the adjusted odds ratio after simultaneously controlling for the potential confounding effects of driver status, sex, region, or era (adjusted OR: 1.02; 95% CI: 0.87, 1.20). When examined together, there was no evidence of effect modification by sex or driver status (*p* = 0.95). When the potential confounders were removed one at a time, no variable changed the injury severity estimate by more than 15% and were concluded to not confound the association between injury severity and age.

**Table 3:** Characteristics of ATV users by injury severity

	Moderate to serious	Mild	Odds Ratio (95% CI)
	*n* = 1826^a^ (38.0%)	*n* = 2983^a^ (62.0%)	
< 16 yrs	1030 (56.4)	1725 (57.8)	0.94 (0.84, 1.06)
Male	1387 (76.0)	2156 (72.3)	**1.21 (1.06, 1.38)**
Driver	1067 (58.4)	1487 (49.8)	**1.30 (1.12, 1.51)**
1990–1995	212 (11.6)	438 (14.7)	1.00
1996–2000	470 (25.7)	758 (25.4)	**1.28 (1.05, 1.57)**
2001–2005	620 (34.0)	869 (29.1)	**1.47 (1.21, 1.79)**
2006–2010	524 (28.7)	917 (30.7)	1.18 (0.97, 1.44)

Note: Injury severity was missing for 196 people who sustained an ATV injury

For all other variables, the reference group is the alternative option (i.e. ≥ 16 yrs, no helmet, passenger, female sex)

Reference group = Mild Injuries

Significantly increased or reduced odds are bolded

^a^ *n* = total number of injuries

Data for head injuries are presented in Table 4. Odds of moderate to serious versus mild head injury were not different in youth compared to adults (crude OR: 0.94; 95% CI: 0.75, 1.19). After simultaneously adjusting for sex, driver status, helmet use, region, and era, the odds of a moderate to serious head injury were not different between youth and adult ATV users (adjusted OR: 1.09; 95% CI: 0.75, 1.60). Sex, driver status, and helmet use did not simultaneously modify the relationship between age and head injury severity (*p* = 0.45). After controlling for helmet use, there was a significantly increased odds of any head injury among youth ATV users compared with adult ATV users (helmet adjusted OR: 1.45; 95% CI: 1.19–1.77). ATV users who wore a helmet were significantly less likely to have a moderate to serious head injury versus a mild injury (crude OR: 0.59; 95% CI: 0.44, 0.78). After adjusting for the confounding effects of driver status, wearing a helmet still significantly reduced the odds of a moderate to serious head injury versus a minor injury (OR: 0.70; 95% CI: 0.52, 0.96).

**Table 4:** Characteristics of ATV users with a head injury by injury severity

	Moderate to Serious	Mild	Odds Ratio (95% CI)
	n* = 592 (49.7%)	n* = 599 (50.3%)	
< 16 yrs-crude	350 (59.1%)	363 (60.6%)	0.94 (0.75, 1.19)
<16 yrs-adjusted^a^			**1.45 (1.19, 1.77)**
Male	422 (70.5%)	421 (71.1%)	1.03 (0.80, 1.33)
Driver	325 (54.9%)	294 (49.1%)	1.25 (0.96, 1.64)
Helmet	161 (27.2%)	228 (38.6%)	**0.59 (0.44, 0.78)**
1990–1995	78 (13.2%)	85 (14.2%)	1.00
1996–2000	160 (27.0%)	165 (27.6%)	1.06 (0.73, 1.54)
2001–2005	191 (32.3%)	180 (30.5%)	1.16 (0.80, 1.67)
2006–2011	163 (27.8%)	169 (28.2%)	1.05 (0.72, 1.53)

*n = total number of injuries

Note: Head injury severity was missing for 54 people who sustained an ATV injury

Reference = Mild injuries

Significantly increased or reduced odds are bolded

^a^adjusted for era, sex, driver status, region and helmet use

For all other variables, the reference group is the alternative option (i.e. ≥ 16 yrs, no helmet, passenger, female sex)

Table 5 identifies risk factors for the most common isolated moderate to serious ATV injuries. In fully adjusted logistic models, youth were more likely to have isolated head injuries versus non-head injuries (OR: 1.45; 95% CI: 1.19–1.77) and fractures versus non-fractures (OR: 1.60; 95% CI: 1.43–1.81) than adults. Helmet use was associated with lower odds of a moderate to serious head injury (OR: 0.35; 95% CI: 0.22–0.55). The odds of an isolated fracture were significantly greater among youth (OR: 1.41; 95% CI: 1.00–1.99) and males (OR: 1.49: 95% CI: 1.02, 2.15).

**Table 5:** Risk factors for the most common isolated moderate to serious ATV-related injuries

Variable^a^	Head Injuries	Fractures	Internal Injuries
	*N* = 559	*N* = 1570	*N* = 61
<16 years	0.85 (0.53, 1.36)	**1.41 (1.00, 1.99)**	0.97 (0.53, 1.81)
Helmet	**0.35 (0.22, 0.55)**		
Driver	0.92 (0.55, 1.55)	1.21 (0.83,1.77)	1.31 (0.62, 2.75)
Male	0.67 (0.41, 1.11)	**1.49 (1.02, 2.15)**	1.89 (0.84, 4.21)
1996-2000^b^	1.24 (0.32, 4.93)	1.55 (0.82, 2.93)	1.13 (0.29, 4.38)
2001-2005^b^	1.25 (0.33, 4.77)	1.47 (0.80, 2.69)	1.73 (0.48, 6.17)
2006-2010^b^	1.12 (0.29, 4.23)	1.39 (0.87, 1.87)	0.86 (0.23, 3.28)

Data are presented as Odds Ratios and 95% CI

For all other variables, the reference group is the alternative option (i.e. ≥ 16 yrs, no helmet, passenger, female sex)

Significantly increased or reduced odds are bolded

^a^Each odds ratio is simultaneously adjusted for the other listed variables

^b^1990-1995 is the reference

## Discussion

The results provide two novel contributions to the area of ATV-related injuries in youth. First, youth less than 16 years of age within the CHIRPP database are at a greater odds of isolated fractures and head injuries, but not at a greater odds of general moderate to serious injuries relative to adults. Second, helmet use is significantly protective of head injuries generally but particularly moderate to serious head injuries.

Policy and expert statements commonly cite legislating a minimum ATV driver’s age as an intervention to curb ATV-related injuries in youth [1, 9]; unfortunately, there has been little empirical evidence to help support such important legislation. A case control study of ATV users in the US, found that the risk of any ATV-related injury was ~12-fold higher (95% CI: 4.6–31.3) in youth ≤15 years, compared with adults >46 years [21]. Furthermore, every additional year of experience riding an ATV reduced the risk of injury by ~4%[21]. We found that while the severity of ATV-related injuries was not greater among riders under 16 years of age, the risk of certain injuries, particularly head injuries and fractures are significantly more common among younger users of ATVs. These data support current calls for legislation to limit use of ATV’s to older adolescents and adults due to an increased risk of certain injuries.

Engine size is an established determinant of ATV-related injuries in youth [10], suggesting that matching engine size to developmental and physical attributes may be a key aspect of ATV-related policies. Previous natural experiments of legislating ATV use have yielded mixed results. While one study did not observe any change in ATV-related injuries in youth from regions in which a minimum driver’s age was legislated [13]; another study found abandoning legislation for a minimum driver’s age resulted in a 5-fold increase in ATV-related hospital trauma (6.9/year to 31.6/year) at a single trauma centre in the United States [22]. The current observation that youth are more likely to suffer from fractures and head injuries than adults supports the argument that younger children are at a greater risk for some injuries when riding an ATV and that there is evidence to support calls for limited use of ATVs among younger children and youth.

Helmets decrease the risk of severe and fatal injuries when riding an ATV or motorcycle [13, 14, 18, 23]. Data on >11,000 hospitalized patients in a national registry in the US, found that unhelmeted riders were 2–3 times more likely to suffer a traumatic brain injury or die from an injury, than helmeted riders [23]. Similar to other studies, we found that younger adolescents and children are more likely to wear helmets [14]. However, we also found that fewer than 50% of children presenting to an emergency department for an ATV-related injury reported wearing a helmet at the time of the injury. In contrast to other studies [24], we found that the proportion of riders wearing a helmet decreased over the past 15 years (71% in 1990–1995 vs 59% in 2000–2005, *p* < 0.01). Among children and adolescents presenting to an emergency department with an ATV-related injury, helmet use reduced the odds of a moderate to serious head injury by nearly 30%. These data support findings from studies of ATV and cycling-related injuries, reinforcing the belief that helmets protect against severe injury in children exposed to high-risk activities [13, 19, 25, 26] and support calls for legislating helmet use for ATV passengers and riders.

A strength of this study is that CHIRPP data represent over 20 years of surveillance of the majority of Canada’s tertiary pediatric care facilities. A unique feature of CHIRPP, and second strength of the study, is the inclusion of injuries that did not require admission, including minor injuries requiring outpatient follow-up or emergency intervention. Despite these strengths, several limitations to the study design should be addressed. First, as with any self-reported instrument, the risk of misclassification bias, particularly for helmet use and driver status, reduces the internal validity of the estimates for these two confounding variables. Second, the data repository is not population-based and older children and those with less serious injuries tend to report to non-tertiary care centres [27]. Third, several variables that may predict moderate to serious injuries were not included in the CHIRPP database, including, ATV-driving experience, terrain where ATV was being used at the time of injury, drugs and alcohol use at the time of injury, parental supervision, and hours logged as either driver or passenger prior to the incident. Fourth, the majority of serious injuries that required direct admission to intensive care facilities were not included in the analysis as parents and physicians typically do not complete the CHIRPP intake form in those instances, reducing the number of serious injuries included in this dataset. Fifth, as a notable portion of data were missing for driver status and helmet use in the database, estimates on the association between these variables and injury severity may be biased if these variables were not missing at random. Finally, data from patients who present directly to a community hospital, were transferred to intensive care, or were fatalities at the scene would not be included in the analysis resulting in an under-representation of serious ATV-related injuries. Despite these limitations, the CHIRPP database has provided important information on injury trends among children and adolescents presenting to emergency departments in Canada [11, 19, 28], that can inform future policies or analyses with more robust datasets.

## Conclusion

Within the CHIRPP database, children less than 16 years of age are at a greater risk of head injuries and fractures, but not moderate to serious injuries following an ATV injury compared to adults. Furthermore, helmet use is associated with significant protection from moderate to serious head injuries in youth. These data support current calls for legislation for a minimum age and mandatory helmet use for children and adolescents riding ATVs.

## Abbreviations

ATV: All-terrain vehicle
CHIRPP: Canadian hospitals injury reporting and prevention program

## References

[1] Yanchar NL: Preventing injuries from all-terrain vehicles. Position Statement. In*.* Canadian Paediatrics Society; 2012. 10.1093/pch/17.9.513 PMC3496361 24179426

[2] Su W, Hui T, Shaw K (2006). All-terrain vehicle injury patterns: are current regulations effective?. J Pediatr Surg.

[3] Yuma PJ, Maxson RT, Brown D (2006). All-terrain vehicles and children: history, injury burden, and prevention strategies. J Pediatr Health Care.

[4] US Consumer Product Safety Commission. Annual report of ATV-related deaths and injuries [https://www.cpsc.gov/s3fs-public/pdfs/atv2009.pdf]. Accessed 16 Mar 2014.

[5] Shults RA, Wiles SD, Vajani M, Helmkamp JC (2005). All-terrain vehicle-related nonfatal injuries among young riders: United States, 2001-2003. Pediatrics.

[6] Collins CL, Smith GA, Comstock RD (2007). Children plus all nonautomobile motorized vehicles (not just all-terrain vehicles) equals injuries. Pediatrics.

[7] Shults RA, West BA, Rudd RA, Helmkamp JC (2013). All-terrain vehicle-related nonfatal injuries among young riders in the United States, 2001-2010. Pediatrics.

[8] Helmkamp JC, Furbee PM, Coben JH, Tadros A (2008). All-terrain vehicle-related hospitalizations in the United States, 2000-2004. Am J Prev Med.

[9] Prevention AAoPCoIaP (2000). All-terrain vehicle injury prevention: two-, three-, and four-wheeled unlicensed motor vehicles. Pediatrics.

[10] Denning GM, Harland KK, Jennissen CA (2014). Age-based risk factors for pediatric ATV-related fatalities. Pediatrics.

[11] Vanlaar W, McAteer H, Brown S, Crain J, McFaull S, Hing MM (2015). Injuries related to off-road vehicles in Canada. Accid Anal Prev.

[12] Lord S, Tator CH, Wells S (2010). Examining Ontario deaths due to all-terrain vehicles, and targets for prevention. Can J Neurol Sci.

[13] McBride AS, Cline DM, Neiberg RH, Westmoreland KD (2011). Pediatric all-terrain vehicle injuries: does legislation make a dent?. Pediatr Emerg Care.

[14] Pelletier JS, McKee J, Ozegovic D, Widder S (2012). Retrospective review of all-terrain vehicle accidents in Alberta. Can J Surg.

[15] Beaudin M, Dunand L, Piche N, Rousseau E, St-Vil D (2014). Legislation in Quebec for all-terrain vehicles: are we doing enough?. Pediatr Emerg Care.

[16] Mackenzie SG, Pless IB (1999). CHIRPP: Canada's principal injury surveillance program. Canadian Hospitals Injury Reporting and Prevention Program. Inj Prev.

[17] Macarthur C, Dougherty G, Pless IB (1997). Reliability and validity of proxy respondent information about childhood injury: an assessment of a Canadian surveillance system. Am J Epidemiol.

[18] Keenan HT, Bratton SL (2004). All-terrain vehicle legislation for children: a comparison of a state with and a state without a helmet law. Pediatrics.

[19] Linn S, Smith D, Sheps S (1998). Epidemiology of bicycle injury, head injury, and helmet use among children in British Columbia: a five year descriptive study. Canadian Hospitals Injury, Reporting and Prevention Program (CHIRPP). Inj Prev.

[20] Canadian Institute for Health Information. National Trauma Registry Analysis in Brief: ATV Injury Hospitalizations in Canada, 2004–2005. Toronto: CIHI; 2007. (https://secure.cihi.ca/free_products/ntr_atv_aib_2007_e.pdf).

[21] Rodgers GB, Adler P (2001). Risk factors for all-terrain vehicle injuries: a national case-control study. Am J Epidemiol.

[22] Fonseca AH, Ochsner MG, Bromberg WJ, Gantt D (2005). All-terrain vehicle injuries: are they dangerous? A 6-year experience at a level I trauma center after legislative regulations expired. Am Surg.

[23] Bowman SM, Aitken ME, Helmkamp JC, Maham SA, Graham CJ (2009). Impact of helmets on injuries to riders of all-terrain vehicles. Inj Prev.

[24] Leblanc JC, Beattie TL, Culligan C (2002). Effect of legislation on the use of bicycle helmets. Cmaj.

[25] Persaud N, Coleman E, Zwolakowski D, Lauwers B, Cass D (2012). Nonuse of bicycle helmets and risk of fatal head injury: a proportional mortality, case-control study. Cmaj.

[26] Wesson DE, Stephens D, Lam K, Parsons D, Spence L, Parkin PC (2008). Trends in pediatric and adult bicycling deaths before and after passage of a bicycle helmet law. Pediatrics.

[27] Macpherson AK, White HL, Mongeon S, Grant VJ, Osmond M, Lipskie T, Mackay MJ (2008). Examining the sensitivity of an injury surveillance program using population-based estimates. Inj Prev.

[28] Yanchar NL, Kennedy R, Russell C (2006). ATVs: motorized toys or vehicles for children?. Inj Prev.

